# EEG brain signals to detect the sleep health of a driver: An automated framework system based on deep learning

**DOI:** 10.3389/fnhum.2022.915276

**Published:** 2022-08-25

**Authors:** Halima Ettahiri, José Manuel Ferrández Vicente, Taoufiq Fechtali

**Affiliations:** ^1^Departamento de Electrónica, Tecnología de Computadores y Proyectos - Campus la Muralla, Universidad Politécnica de Cartagena, Cartagena, Spain; ^2^Department de Biosciences, Exploration Fonctionnelle Intégrée, Faculté de Sciences et Techniques de Mohammedia, Université Hassan II Casablanca, Mohammedia, Morocco

**Keywords:** mental fatigue, sleepy, normal, EEG signals, CNN, deep learning

## Abstract

Mental fatigue is complex disorganization that affects the human being's efficiency in work and daily activities (e.g., driving, exercising). Encephalography is routinely used to discern this fatigue. Several automatic procedures have deployed conventional approaches to support neurologists in mental fatigue detection episodes (e.g., sleepy vs. normal). In all of the traditional procedures (e.g., support vector machine, discrimination fisher, K-nearest neighbor, and Bayesian classification), only a low accuracy is achieved when a binary classification task (e.g., tired vs. normal) is applied. The convolutional neural network model identifies the correct mathematical manipulation to turn the input into the output. In this study, a convolutional neural network is trained to recognize brain signals recorded by a wearable encephalographic cap. Unfortunately, the convolutional neural network works with large datasets. To overcome this problem, an augmentation scheme for a convolutional neural network model is essential because it can achieve higher accuracy than the traditional classifiers. The results show that our model achieved 97.3% compared to the state-of-the-art traditional methods (e.g., SVM and LDA).

## 1. Introduction

Mental fatigue is neurological disorganization that affects a driver's behavior. Electroencephalography (EEG) is a powerful and non-invasive method that is commonly used to track brain activity and detect mental fatigue. EEG records are examined by neurologists to diagnose and classify sleep disorder samples. However, visual exploration is laborious and requires an expert neurologist. Moreover, the examination of EEG records reduces the effectiveness of an expert (Ullah et al., 2018). All of these restrictions have inspired researchers to develop and design an automated system to help neurologists to categorize normal and sleepy EEG brain signals (Zammouri et al., 2018).

Recently, a large amount of research has attempted to distinguish between the signals of sleepy and normal subjects. Generally, the data quantity in this type of classification is not good enough to train a classifier due to the lack of experiments. In addition, the presence of noise in s can generate difficulties in learning the brain patterns that are associated with normal and sleepy cases.

The current automatic techniques that are used to detect mental fatigue use the traditional algorithms of ML, such as Support Vector Machines (SVM) or Latent Dirichlet Allocation (LDA) (Ettahiri and Fechtali, 2020), can reach high accuracy for one problem but can fail in other cases. This difficulty depends on many things, especially from the fact that labeled data is less available. To assist and help neurologists, a general automatic system design is proposed and shows high performance, despite the small number of training samples.

Experts have presented frameworks to detect the state of drowsiness using features extracted from EEG signals by hand-designing methods. Some of them used spectral and temporal aspects from EEG signals, while others tend to use a classifier based on the Short-Time Fourier Transformation (STFT) (Zammouri et al., 2018). Meanwhile, the Deep Learning (DL) approach codes different numbers of features that are not related and adapted to the dataset, which demonstrates very good results in this application. Furthermore, the features pulled out by the DL have shown to be more robust and vigorous than the traditional methods. Consequently, to improve the accuracy of this experience of the driver's sleep health, our proposed method is based on DL.

Recently, various DL methods have been shown to have a good performance in many different field applications. Many applications use CNN for image recognition and the Deep Neural Network (DNN) is used to understand music generation and text readability. The DL method is used to avoid the selection of adequate feature combinations. Although traditional methods are fast to train datasets compared to the DL method, the only obstacle is the data that is needed to train one model. An optimal approach to training the deep model is to apply scheme augmentation.

The major contributions of this experiment are dataset preparation and the system architecture, which is based on DNN for binary EEG signal classification. The rest of this article is structured as follows. A review of the literature is presented in Section 2. Section 3 describes the proposed framework model and data augmentation. The results are presented and discussed in Section 4. Finally, the conclusion is given in Section 5.

## 2. Literature review

Knowledge of a driver's sleep health is a complex classification procedure, the features are extracted and then classification algorithms are applied. In this section, the state-of-the-art of traditional techniques that are generally used to classify this type of data will be reviewed. In previous studies, the SVM method gives a slightly lower accuracy of between 75 and 88%, while the Latent Dirichlet Allocation (LDA) and k-Nearest Neighbors (k-NN) classifiers provide notably lower accuracies of between 65 and 76.7% and 64.2 and 67.1%, respectively (Ettahiri and Fechtali, 2020). Meanwhile, Primary Domain Controller (PDC) networks result in better classification accuracy than LDA and k-NN in all cases. Moreover, focusing on SVM (Dimitrakopoulos et al., 2017), the best performance is achieved by a PDC metric with an accuracy of 84.7% using 21 features (Boracchi et al., 2017). Similarly, PDC networks had a high performance in terms of accuracy compared to other classifiers.

A recapitulation of all of the traditional methods to classify EEG datasets is applied in our study. The Linear Discriminant Analysis (LDAn) classifier was employed to classify these features and reached a higher accuracy of 87% for normal vs. sleepy, while the SVM classifier achieved the maximum accuracy (i.e., 88% for normal vs. sleepy). The results indicate that 88% precision is attained. In the majority of recent papers, SVM is the most commonly used classifier to distinguish between sleepy and normal volunteers. However, the LDA shows a better outcome when just a single volunteer is trained and tested, providing 87.7% overall accuracy. When doing the generalization for all volunteers, it gives 76.5% of overall accuracy (lower than SVM classification). Most feature extraction techniques are designed by hand and are not adapted to the data. Thus, to ameliorate the accuracy and the precision of a sleep health detection system, the DL approach is used to avoid the need for traditional feature extractors and classifiers. So far, the DL method has rarely been used for mental fatigue detection because of the small amount of available data. Consequently, a DL approach proposes a deep model that involves a small number of learnable parameters and then efficiently classifies EEG brain signals as a normal or sleepy person.

This systematic literature review has synthesized and recapitulated the published works related to deep classification methods for sleep detection for drivers. Mendonca et al. (2018) found that for a single source sensor, ECG signals result in the highest global classification. However, sleep apnea is a disease that is related to respiration. Thus, signals due to the use of public datasets that are less affected by noise give higher accuracy with ECG (Mendonca et al., 2018).

Craik et al. (2019) described the current practice of DL in EEG classification. Meanwhile, Zeng et al. (2018) predicted mental state using two classification models (called EEG-Conv and EEG-Conv-R). In fact, the DL models that are used to detect the mental state and the EEG make it easier and more precise to define the state of the brain.

## 3. Proposed framework

### 3.1. Creativity and innovation

In the past decades, there have been numerous advancements in the field of technology. Since the world's problems are increasing in complexity as it progresses, it is imperative that advances in science and technology be made in areas such as automatic recognition and detection. One of these problems is mental fatigue, which contributes to many accidents around the world. In a driving environment, it is necessary that fatigue detection is performed in a non-intrusive way and that the driver is not bothered with alarms when he or she is not drowsy. Using machine learning and deep learning, we provide a different method to comprehend the meaning of fatigue, its detrimental impacts, and strategies to detect fatigue. The work also discusses classifier performance measures and comparison analyses with different automatic detection using the EEG signals. In fact, the fatigue detection requires a lot of analysis, and especially the analysis of EEG signals, these signals are difficult to provide and very expensive and depend on several parameters and many conditions, the first part of this work was very difficult since we made a lot of time to find our volunteers and to succeed in this experience, for the part of the analysis using the different algorithms of deep learning and trying to adapt them to our data. Therefore, besides proposing a real data base of EEG signals, we can also mention the adaptation of our data to the model CNN, also the augmentation the data which we detailed in the next chapter to get good results.

### 3.2. Laboratory experience

Before applying any application of the model, the main idea of this experiment and the dataset that was used need to be made clear. In this experiment, the impact of sleep deprivation on mental fatigue is assessed (Daniela et al., 2010). In total, 20 volunteers were selected to participate in this study, none of them had a medical history, neurological or psychiatric disorders, or drug addiction (Ferrara et al., 2006; Tempesta et al., 2016). The participants were randomly assigned to one of two groups: (i) the normal sleep group and (ii) the deprivation sleep group (Ferrara et al., 2006). EEG signals were collected using the Open VIBE interface, which was developed by INRIA, and ENOBIO 8 cap material was used.

EEG signals were collected from 20 people using ENOBIO 8, which allows us to perform tests in three sessions, each session's duration is 7 min (total 21 min per volunteer), with 40 trials in each session. Therefore, the EEG signal analysis is important to get conclusions for different volunteer groups. The EEG signals are able to define mental fatigue and its impact. The DL (CNN model) is adapted to the problem of EEG classification and then compared to those obtained using SVM and linear discriminant analysis.

### 3.3. Participants

Five healthy men and five healthy women aged between 26 and 36 years old participated voluntarily in the first part of this experiment. In the second part, 10 volunteers from different countries took part in the study, aged between 25 and 30 years old. In the two experiment groups, the volunteers were informed of the experiment. No ethical review or approval was required for this study and none of the subjects suffered from any mental illnesses or disorders that can disturb the collected results.

The volunteers were selected by recruiting from different genders. To get better EEG signals, only measured signals without noise are considered. Sometimes, noise can appear because of cosmetic products, hair gel, and curly hair. In addition, the age range is important to suit the case drivers.

### 3.4. Data acquisition

**Figure 1:**
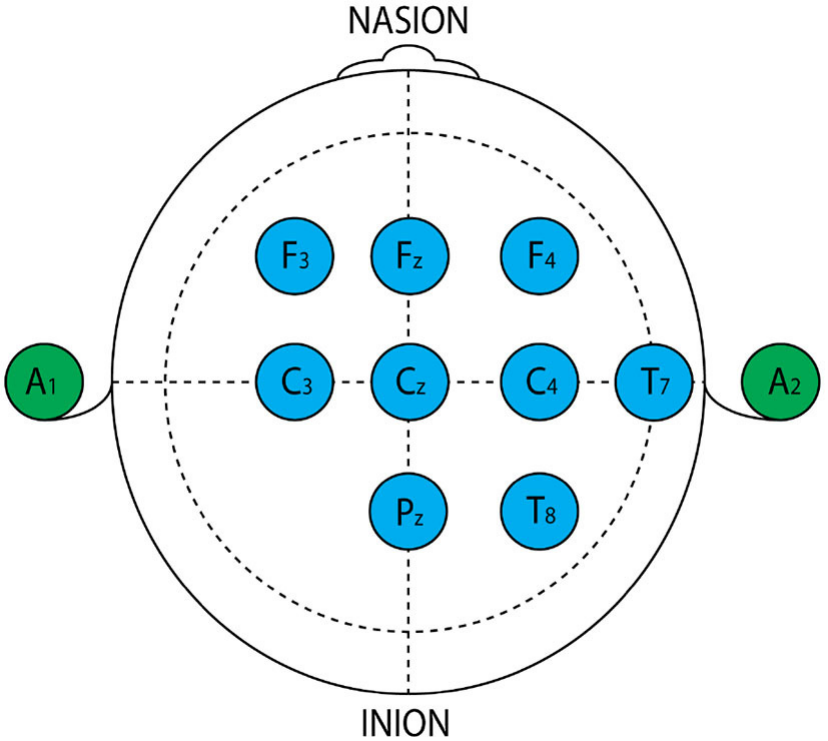
Placement of the electrodes. Blue color: electrodes used in our experiment.

EEG data were recorded in the SICOMO laboratory (Polytechnic University of Cartagena, Spain) and the OpenVibe acquisition protocol was respected. The EEG headset with eight electrodes was placed as shown in Figure 1.

In the first group (normal subject) the apparition of alpha waves (Figure 1) shows that the subject is in a calm state, that waves normally appear in the resting mood, meditative state for the brain. While the Beta waves are needed to react and make decisions, Alpha waves help in absorbing new information, overall mental coordination, remaining alert yet calm, mind/body integration, and learning.

The sampling frequency was set at 500 Hz. The EEG signals recorded are filtered using a pass-band filter [1–30 Hz]. The purpose of a filter is to eliminate noise components from signals. The distinction between noise and signal would be simple if the signal was well-known. In this work, the original signals arrive simultaneously to the eight electrodes. The signal, in this case, can be expressed as follows:


(1) $$\begin{array}{r} X=AS+N \end{array}$$

Where *X* = [*X*1, …., *XN*] is the signal matrix and *S* = [*S*1, …, *SN*] is the original data.

### 3.5. Problem statement

The acquisition system generating EEG signals is the original of our dataset which is shown in Figure 1 is characterized by a reel data made in the laboratory. Furthermore, our dataset is not huge in quantity but with big size dimension signals and each signal has mono or multi sub signals with different dimensions and quantities. Seeing that, the detection of mental fatigue is a complex, challenging mission. The classification mission often used feature extraction methods that have been proven to be effective for different object recognition tasks. Indeed, deep learning reduces this phase by automating the learning and extracting the features from the specific architecture in the network. Major problems limiting the use of deep learning methods are the availability of computing power and training data. Training a convolutional network from start to finish on such hardware of ordinary consumer laptops and with the dataset's size would be enormously time consuming.

**Figure 2:**
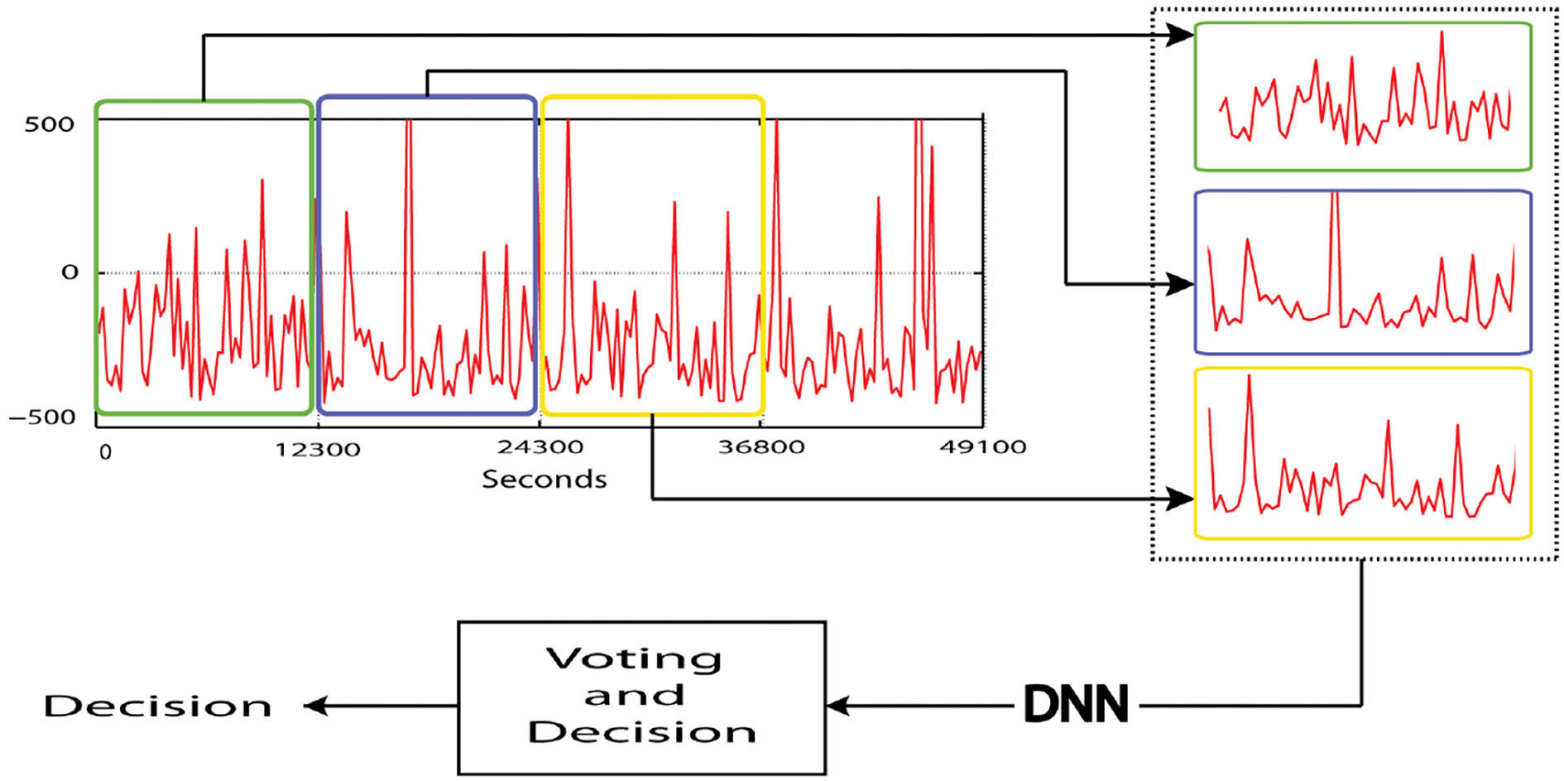
Division of the signal architecture.

In this work, we had access to a high graphics processor applied for the search goal. Moreover, convolution networks need a wide quantity of medium-sized training data. As the collection and recording of a sufficiently large dataset are hard-working all works in this topic focus on ready datasets. This is a problem because we did not have an available benchmark of EEG signals of pipeline signals that is pretrained previously and we do not have the same EEG signals quality. For the same reasons, we construct data ourselves from the few data we have of EEG signals by cutting and flipping horizontal and vertical, rotating, or subsampling with different crops and scales which is detailed in the part (Data Augmentation). The general step of the proposed method is detailed illustrating the adjustment process in Algorithm 1 and Figure 2.

**Algorithm 1:** **Prepossessing and training process**.

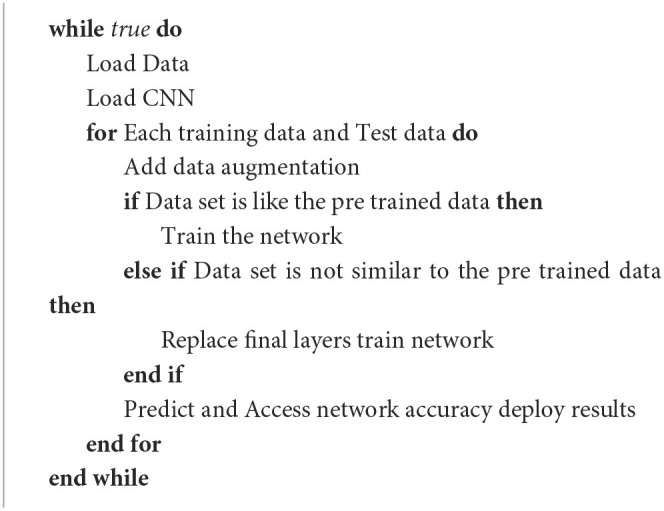

### 3.6. Overview of convolutional network structure

**Figure 3:**
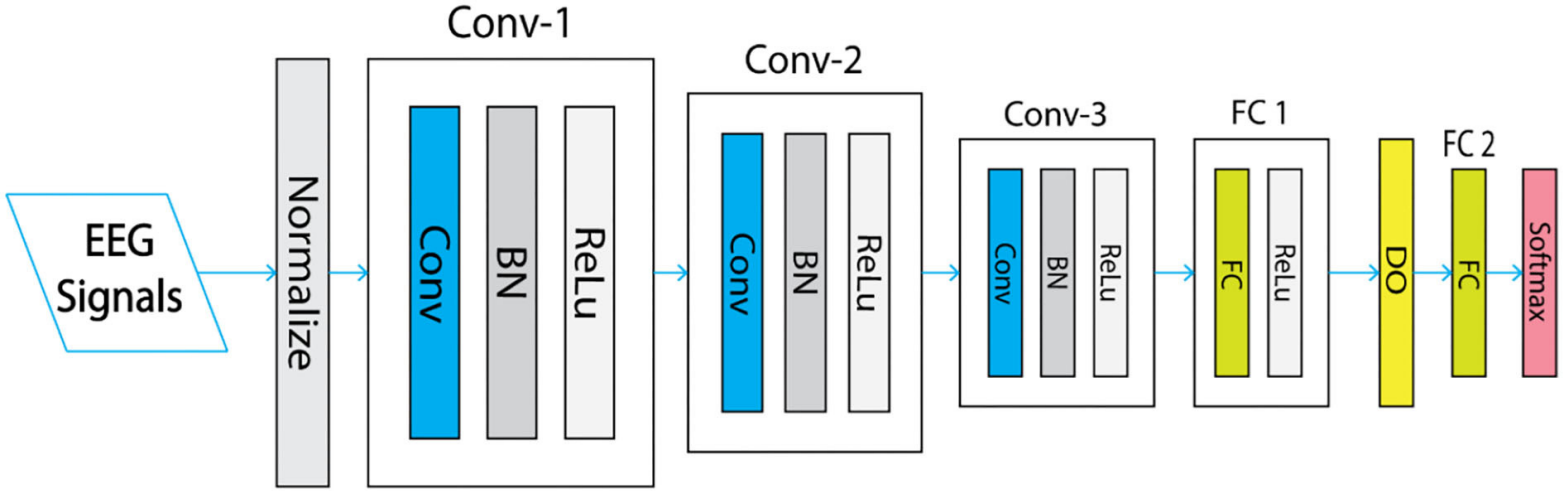
1 P convolutional neural network (CNN) architecture.

The linear and non-linear processes have been implicated with the convolutional neural model which is a set of overlapping layers. The CNN head structure blocks are constituted by the convolutional layer, cluster layer, rectified linear units (ReLU) layer linked to a fully connected layer and a loss layer bottom (Figure 3).

#### 3.6.1. The detailed network architecture

Convolutional neural network is one of the pretrained system based on a deep learning system with a specific architecture. Three convolutional layers constitute the net with core sizes 5*5, 3*3, and 3*3 for each convolutional Conv1, Conv2, Conv3, respectively, considering the specificality of the structure of the files in the dataset.

The resolution related to the first Convolutional layer is 227*227. It is stated to have 96 cores with a stride of 4 pixels and a size of 11*11. In total, 256 kernels with a stride of 1 pixel and a size of 5*5 are stacked in the second convolutional layer and filtered from the pooling output of the first convolutional layer. The output of the previous layer is connected to the remainder of convolutional layer with a stride of 1 pixel for each convolutional layer with 384, 384, and 256 kernels of size 3*3 and without pooling grouping. The following layer is piled to 4,096 neurons for each fully connected layers and a max-pooling layer. After all, the last fully connected layer's output is powered by SoftMax which generates a two class label sharing out. In this architecture, a max pooling layer is piled with 32 pixels size and the stride of 2 pixels only after the two beginning and the fifth convolutional layers. The application of ReLU non-linearity activation function layer in each fully connected instead of sigmoid and Tanh activation functions improve the speed of convergence. A full network requirement and the principal parameters of the CNN design are presented in the following Table 1 and detailed in the third section.

**Table 1:** The principal parameters of the convolutional neural network (CNN) design.

1	Data	CSV input	39*39*3 CSV with zero center normalization
2	Conv1	Convolution	convolution with stride [4 4] and padding [0 0 0 0]
3	Relu1	ReLU	ReLU
4	Norm1	Cross channel normalization	Cross channel normalization with 5 channels per element
5	Pool1	Max pooling	3*3 max pooling with stride [2 2] and padding [ 0 0 0 0]
6	Conv2	Convolution	256 5*5*48 convolutions with stride [1 1] and padding [2 2 2 2]
7	Relu2	ReLU	ReLU
8	Norm2	Cross channel normalization	Cross channel normalization with 5 channels per element
9	Pool2	Max pooling	3*3 max pooling with stride [2 2] and padding [ 0 0 0 0]
10	Conv3	Convolution	348 3*3*256 convolutions with stride [1 1] and padding [1 1 1 1]
11	Relu3	ReLU	ReLU
24	Prob	Softmax	Softmax
25	Output	Classification output	Cross entropy

### 3.7. Data augmentation implementation and layers results

#### 3.7.1. Data augmentation experimental study

In deep learning works, we need an enormous amount of dataset to escape from many problems of unnecessary learning. Below diverse uses, changing the image geometrically is applied to raise this amount of data, two types of data augmentation are used: image translations and horizontal reflections and altering the intensities of the RGB channels. In our case, those methods are not suitable for enlarging our EEG data. Compared with the image, the EEG signal is a continuous signal that changes over time. Regardless, the performance of the feature extraction, the features still are a time series. Consequently, the rotation or shifting of the EEG data of the feature on the time domain will be destroyed. To avoid this issue, we prefer to use the noise addition method to augment the EEG samples. In theory, there are many ways to add noises in the EEG data like (Gaussian, Poisson, Salt, Pepper, etc.). Therefore some of the EEG signals has a very strong randomness and non-stationary behavior. If we add a randomly some local noises, such as Poisson noise, Salt noise, or Pepper noise, which will change the features of EEG data locally. Based on these considerations, in our work, it might make more sense to multiply our signal by a noise array (centered around 1), rather than adding a noise array, we focus on adding noise generated from the multiplication to each feature sample of the original training data to obtain new training samples. The probability density function P of a Gaussian random variable z is defined by


(2) $$\begin{array}{r} P_{G}\left(z\right)=\frac{1}{\sigma \sqrt{2\pi }}e^{\frac{-\left(z-\mu \right)}{2\sigma^{2}}} \end{array}$$

### 3.8. Layers fine tuning experimental results

For classification means, transfer learning methods use new deep grids to leverage information from the preliminary test delivered by a pretrained network to apply it to new patterns in new data. Usually, the training of data from scratch is slower than using the transfer learning and fine-tuning method.

So, using these types of networks allows us to pick up new works without configuring a new network and with a great graphics processor. We evaluated the network by training on EEG signals. For test, 20% of the two categories were chosen as the test and validation datasets, and the rest is the training dataset (80%). Two categories are applied for experiments, and their mean classification accuracy was taken as the results. The signals employed with fixed resolution size as the input of the network would convolve and pool the activations repeatedly, then forward the results into the fully-connected layers and classify the data stream into 2 categories. To prevent decreasing error caused by the low amount of data, the initial learning rate base-lr is set as 0.001. Furthermore, the soft max output layer Fc8 is characterized by 2 categories and the hidden layers Fc6 and Fc7 are piled with 4,096 neurons. Inaccuracy of predictions in classification is presented by the loss function (LF) which measures the optimal strategy. The system is performing well according to the smallest value of LF. As can be seen in Table 2, after 80 iterations, the loss curve tends to zero while the classification accuracy curve tends to 1 which meets the requirements of the optimization objectives. The validation classification accuracy reaches as high as 0.65 when the iteration is 1 while increases to 1 when the iteration is 80 and, the actual test accuracy in Table 1 shows the effect of fine tuning layers of the network, in the form of 1p CNN algorithm, where the network will be fine-tuned from layer 6 to layer 8 and all the previous layers are kept constant with no update.

**Table 2:** Training results for the first epoch.

**Epoch**	**Iteration**	**Time elapsed HH:MM:SS**	**Validation accuracy (%)**	**Validation loss**	**Base learning rate**
1	1	00:00:10	67.47	1.0146	0.0010
1	3	00:00:15	70.19	0.6706	0.0010
1	6	00:00:16	75.77	0.3102	0.0010
1	9	00:00:16	88.37	0.3085	0.0010
1	12	00:00:17	97.09	0.2529	0.0010

### 3.9. Evaluation of the model proposed with k-Fold cross-validation

The neural network model has to be evaluated by scikit-learn for training data, which is able to estimate the models employing multiple procedures (Andrzejak et al., 2001; Brownlee, 2017). Meanwhile, k-fold cross-validation is commonly used to evaluate ML models. The model evaluation procedure architecture is first explained. In this case, the number of folds is fixed at 10 (an excellent by default). Then, the data are mixed before partitioning them (shuffle the data). Thus, the evaluation of the model (estimator) on the dataset deploys a 10-fold procedure (k-fold). The evaluation of the model takes approximately 10 s, and it returns an object that helps to describe the evaluation of the 10 constructed models for each dataset split.


(3) $$\begin{array}{l} k_{fold}=K_{Fold}(n\_splits=k,shuffle=True,random\_state\\ \;=seed)\;k=10; \end{array}$$

The results obtained and the SDs are regrouped for model accuracy in the dataset. This problem has a reasonable estimation of the performance of the model on unseen data, also within the realm of known top results.

## 4. Discussion

The EEG signals (i.e., normal vs. sleepy) involved a lot of algorithms to test the classification in binary problems when comparing it with the state-of-the-art methods (Khan et al., 2012; Hussain et al., 2016). To the best of the author's knowledge, the DL method has never been used for this type of problem. This classification consists of extracting the discriminatory features from EEG signals and thus accomplishing classification. A general review of all state-of-the-art related techniques is provided, which uses many feature extraction and classification methods for mental fatigue classification from the extracted EEG signals. The SVM classifier was employed in a previous study to classify these features with the same dataset to achieve maximum accuracy. The maximum accuracy achieved for the subjects is 88.7%. With another classifier, TREE, the achieved accuracy was 75.77% for the same dataset.

**Figure 4:**
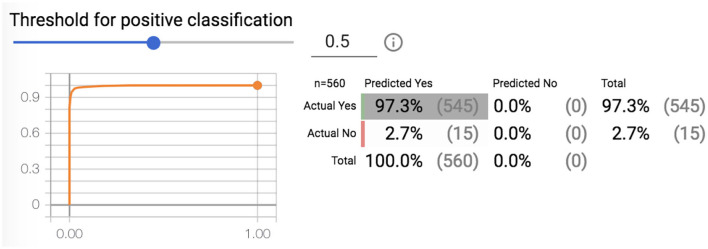
Threshold for positive classification.

This shows the vigor of this proposed framework based on 1-D CNN and indicates that it has a greater stimulus than the other methods. The accuracy achieved with the proposed system is 97.33% for the dataset for the 20 volunteers, as shown in Figure 4.

Previous work has proposed framework systems for sleep detection for drivers (Ettahiri and Fechtali, 2020). The recognition of mental fatigue was classified by SVM (88.7 %), TREE(78%), LDA(87%), and KNN (86%). Table 3 represents a comparison in term of the accuracy corresponding to each method. The maximum accuracy was detected in our subjects with the SVM classifier. In comparison, with the proposed method, we achieved a maximum (Table 4) accuracy of 97.3%, which demonstrates the efficiency of the DL compared to the traditional methods.

**Table 3:** Accuracies using LDA, SVM, KNN, and TREE classifiers.

	**Tree**	**LDA**	**SVM**	**KNN**
BP	0.7875	0.8750	0.8875	0.7875
CSP	0.9125	0.9375	0.9250	0.8375
BP+CSP	0.9500	0.9375	0.9250	0.8625

**Table 4:** Metric method to evaluate the model.

**Class**	**Accuracy**	**Precision**	**F1-score**
0	0.97	0.91	0.87
1	0.89	0.85	0.88

In other comparisons of different approaches, the authors used a publicly available dataset, thus they were able to provide a fair comparison of different approaches. The CNN approach was compared with one research based approach on the LSTM network and seven feature-based research studies (Jiao et al., 2020). The best accuracy was obtained with their proposed method, while the LSTM method had a slightly lower accuracy. On average, all seven feature-based approaches had more than 5% lower accuracy. The goal of this study (Chen et al., 2018) is to propose a comprehensive approach based on EEG signals to explore whether FBN changes from the alert state to the drowsy state and to find out ideal neurophysiology indicators to detect driver drowsiness in terms of FBN. Based on this, two functional brain network (FBN) approaches, SL and MST, are first combined and applied to feature recognition and classification. For classification, these brain network features are fed into four classifiers considered namely support vector machines (SVM), K nearest neighbors classifier (KNN), logistic regression (LR), and decision trees (DT) (Min et al., 2017).

**Figure 5:**
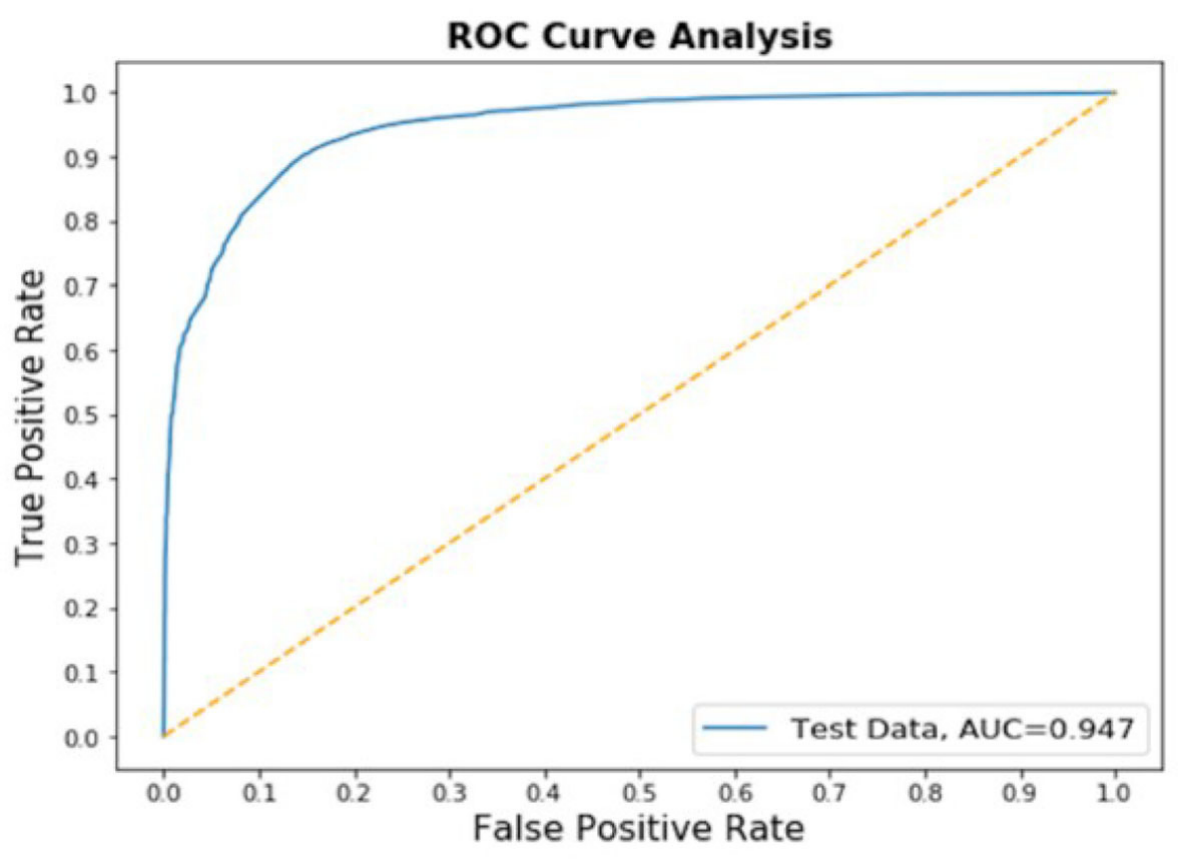
The ROC of the model.

We have added other metrics methods (Figure 5).

## 5. Conclusion

This paper proposes an automatic framework system to detect driver sleep fatigue (sleepy and normal). The suggested model is based on DL. For this framework, a model based on CNN is introduced that uses a minimum number of learnable parameters (electrodes). The proposed model has an ensemble of CNN models that include EEG signals as an input, which are divided into classified sub-signals by CNN models. Finally, to deal with the small size of the dataset, the augmentation scheme has been introduced into the CNN framework. The CNN framework is achieved and trained without great effort on chips where memory is limited. The proposed system has a good performance with a small dataset and few parameters. It will help neurologists and experts to detect the state of the brain when it feels sleepy.

## Data availability statement

The original contributions presented in the study are included in the article/supplementary material, further inquiries can be directed to the corresponding author.

## Ethics statement

Ethical review and approval was not required for the study on human participants in accordance with the local legislation and institutional requirements. Written informed consent from the participants was not required to participate in this study in accordance with the national legislation and the institutional requirements.

## Author contributions

HE and JF conceived of the presented idea. HE, JF, and TF developed the theory and performed the computations. JF encouraged HE to investigate about deep learning and supervised the findings of this work. All authors discussed the results and contributed to the final manuscript.

## Conflict of interest

The authors declare that the research was conducted in the absence of any commercial or financial relationships that could be construed as a potential conflict of interest.

## Publisher's note

All claims expressed in this article are solely those of the authors and do not necessarily represent those of their affiliated organizations, or those of the publisher, the editors and the reviewers. Any product that may be evaluated in this article, or claim that may be made by its manufacturer, is not guaranteed or endorsed by the publisher.
